# Lymphocytic Choriomeningitis Virus Infection, Australia

**DOI:** 10.3201/eid2808.220119

**Published:** 2022-08

**Authors:** Leon Caly, Ashleigh F. Porter, Joanna Chua, James P. Collet, Julian D. Druce, Michael G. Catton, Sebastian Duchene

**Affiliations:** Monash University, Clayton, Victoria, Australia (L. Caly);; The Peter Doherty Institute of Infection and Immunity, Melbourne, Victoria, Australia (L. Caly, A.F. Porter, J. Chua, J.D. Druce, M.G. Catton, S. Duchene);; University of Sydney, Sydney, New South Wales, Australia (J.P. Collet);; Dubbo Hospital, Dubbo, New South Wales, Australia (J.P. Collet)

**Keywords:** lymphocytic choriomeningitis virus, genome sequencing, Australia, viruses, zoonoses

## Abstract

During a mouse plague in early 2021, a farmer from New South Wales, Australia, sought treatment for aseptic meningitis and was subsequently diagnosed with locally acquired lymphocytic choriomeningitis virus infection. Whole-genome sequencing identified a divergent and geographically distinct lymphocytic choriomeningitis virus strain compared with other published sequences.

A member of the Arenaviridae family, lymphocytic choriomeningitis virus (LCMV) is an enveloped virus comprised of a bisegmented (large [L] and small [S]), negative-stranded RNA genome encoding 2 polypeptides per segment. First discovered in the 1930s during a study of epidemic encephalitis in St. Louis, Missouri, USA (*1*), LCMV is presumed now to be localized to all continents (excluding Antarctica) based on the distribution of its primary host, the common house mouse (*Mus musculus*). In early 2021, a mouse plague started in western New South Wales, Australia, and spread to the adjoining jurisdictions of Queensland, Victoria, and South Australia, causing considerable losses to the Australia agricultural and grain industry. We report a case of acute LCMV infection in a male farmer from New South Wales.

Although LCMV is considered a truly global virus, acute LCMV infection is rarely diagnosed, possibly because most infected, immunocompetent patients have mild, self-limiting symptoms, such as headache, fever, and myalgia, or are completely asymptomatic and thus never seek treatment. In rare instances, patients have onset of aseptic meningitis or meningoencephalitis but usually recover with no sequalae (*2*). Furthermore, LCMV is not routinely considered as part of a differential diagnosis (outside of a mouse plague), and testing is not widely available. Fatal infections are rare but have been associated with organ transplantation (*3*). LCMV infection in pregnant women has been associated with pregnancy loss and permanent congenital neurologic malformations and chorioretinitis (*4*). Sampling and subsequent genomic sequencing occur only sporadically, usually during a marked spillover event from the reservoir rodent host to humans (*5*).

In early 2021, a 51-year-old male farmer in New South Wales, Australia, sought treatment for headache, neck stiffness, photophobia, and a lower abdominal rash. The patient described a month-long history of arthralgia affecting his low back, left hip, and knee. Serologic and PCR tests were negative for *Neisseria meningitidis*, *Streptococcus pneumoniae,* herpes simplex viruses, enterovirus, parechovirus, cytomegalovirus, and varicella zoster virus. Given the patient’s high zoonotic exposure as a cattle and horse farmer and his recent contact with mice, their carcasses, and droppings (all occurring during a concurrent regional mouse plague), we conducted PCR testing for LCMV on a sample of cerebrospinal fluid, which returned a positive result (*6*). With supportive care, the patient’s symptoms resolved, and he was discharged and remains well.

After the patient’s diagnosis, we sought to determine the evolutionary origin of this specimen in a global context. From 50 μL of the patient’s cerebrospinal fluid, we obtained an almost complete genome sequence for both the L and S viral genome segments by using a nontargeted, sequence-independent, single-primer amplification strategy prior to Illumina library preparation (https://www.illumina.com) (*7*). We estimated a highest clade credibility phylogenetic tree from both S and L (GenBank accession nos. OK356607 and OK356608) gene segments of the LCMV genome (Figure). Our Australia sample was highly divergent (≈75% nucleotide identity) compared with other S and L gene sequences within GenBank. Bayesian molecular dating of the sequence estimated a divergence from the most common recent ancestor in the mid-16th century, with a mean value of 488 years before present (95% highest posterior density 477.9–531.2) for the S segment and a mean value of 443 years (95% highest posterior density 416.2–470.0) for the L segment. Determining the closest relative to our strain is difficult because of a low representation and diversity of complete LCMV genomes worldwide; there is a high bias toward sequences from the United States and China and a gross underrepresentation of strains from Europe. 

**Figure:**
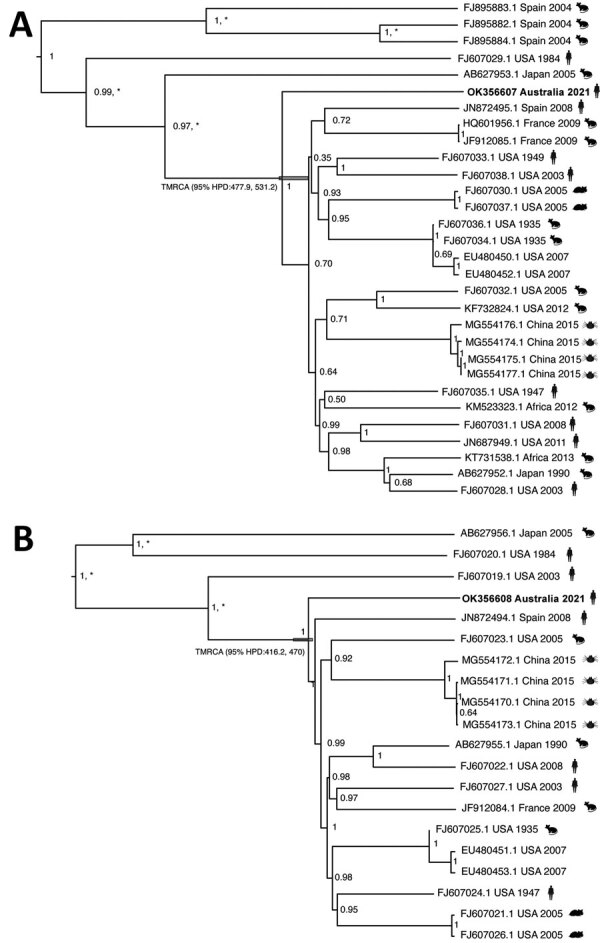
Phylogenetic relationships of a strain of lymphocytic choriomeningitis virus from a man in Australia and the broader lymphocytic choriomeningitis virus phylogeny. Tips are labeled with GenBank sequence accession number, country of origin, year of collection, and host (mice, hamsters, humans, ticks). Trees were generated by using BEAST 1.10.4 (*9*) to estimate the time to the most recent common ancestor between the novel virus sequence and its closest phylogenetic relative. We used the Hasegawa-Kishino-Yano plus gamma substitution model with a strict clock and an exponential growth coalescent tree prior. Because the dataset exhibits high sequence divergence, we calibrated the molecular clock by using previous independent estimates of the substitution rate, with a fixed clock rate for the long segment of 3.7 × 10^–4^ substitutions/site/year and 3.3 × 10^–4^ substitutions/site/year for the short segment (*10*). Highest clade credibility tree of the short segment (GenBank accession no. OK356607) sequences (n = 29) (A) and highest clade credibility tree of the long segment (GenBank accession no. OK356608) sequences (n = 19) (B). Node labels denote the posterior support, and an asterisk represents a bootstrap percentage of >70% support for a specific clade, using 1,000 ultra-fast bootstrap replicates in a maximum-likelihood tree approach using IQ-TREE2 (*11*). The 95% highest posterior density for the divergence time before present of the Australia sample is annotated in the respective node.

LCMV may have been introduced to Australia with the arrival of feral rodents brought by the Dutch and Spanish (17th century) or the French and British (18th century) (*8*). It makes sense, then, that the ancestor of our gene sequences might have been brought to Australia during early European exploration and colonization. This hypothesis can be tested only by monitoring occurrences and gathering more samples of LCMV in rodent populations in both Europe and Australia.
